# Laparoscopic Transduodenal Mucosal Resection with Selective Full-Thickness Excision for Duodenal Duplication in a Child: A Case Report

**DOI:** 10.70352/scrj.cr.26-0382

**Published:** 2026-09-17

**Authors:** Hiroki Ishii, Takahisa Tainaka, Chiyoe Shirota, Satoshi Makita, Masamune Okamoto, Akihiro Yasui, Aitaro Takimoto, Shunya Takada, Kaito Hayashi, Daiki Kato, Hajime Asai, Kazuki Ota, Yui Murata, Liu Jiahui, Guo Yaohui, Hiroo Uchida

**Affiliations:** Department of Pediatric Surgery, Nagoya University Graduate School of Medicine, Nagoya, Aichi, Japan

**Keywords:** duodenal duplication, duodenum, laparoscopy, intraoperative ultrasonography, mucosal resection, pediatric surgery

## Abstract

**INTRODUCTION:**

Duodenal duplication is a rare congenital anomaly with nonspecific symptoms, and preoperative diagnosis is often difficult. Complete resection is recommended, but lesions adjacent to the bile or pancreatic duct are challenging to treat safely in pediatric patients. We report a pediatric case of duodenal duplication successfully managed by laparoscopic mucosal resection with selective full-thickness dissection under intraoperative ultrasonographic guidance.

**CASE PRESENTATION:**

A 5-year-old girl presented with abdominal pain and vomiting. CT revealed a cystic lesion in the pancreatic head region, and the initial differential diagnosis included a pancreatic tumor, pancreatic pseudocyst, choledochal cyst, and lymphangioma. Endoscopic retrograde cholangiopancreatography (ERCP) and endoscopic ultrasonography (EUS) showed no communication between the lesion and the pancreaticobiliary system. Although a solid pseudopapillary neoplasm was initially suspected, repeat EUS demonstrated a characteristic double-layered cyst wall, leading to the preoperative diagnosis of duodenal duplication. Because intermittent abdominal pain persisted and recurrent pancreatitis was suspected, surgical treatment was planned. Laparoscopic surgery was performed using intraoperative ultrasonography to localize the lesion, which was not identifiable on the duodenal serosal surface. After transduodenal exposure, the lesion was dissected from the pancreatic side with full-thickness excision where a safe plane was identified, while only the mucosal layer was removed at the shared wall with the native duodenum to preserve the muscular layer. The duodenum was closed primarily. Histopathological examination confirmed duodenal duplication without malignancy. Postoperatively, she developed a pancreatic fistula caused by peripheral pancreatic duct injury, which was successfully managed by temporary pancreatic duct stenting. She was discharged on POD 19 and remained asymptomatic without recurrence at 1 year.

**CONCLUSIONS:**

Laparoscopic mucosal resection with selective full-thickness dissection under intraoperative ultrasonographic guidance may be a feasible organ-preserving option for selected pediatric patients with duodenal duplication near the pancreatic head. This case highlights the usefulness of intraoperative ultrasonography for localizing a non-serosal duodenal duplication and tailoring the extent of resection. However, pancreatic-side dissection may cause peripheral pancreatic duct injury, and surgeons should be prepared for postoperative endoscopic management when selecting this organ-preserving approach.

## Abbreviations

EUS
: endoscopic ultrasonography
ERCP
: endoscopic retrograde cholangiopancreatography
LECS
: laparoscopic and endoscopic cooperative surgery
MRCP
: magnetic resonance cholangiopancreatography

## INTRODUCTION

Duodenal duplication is a rare congenital anomaly that accounts for approximately 6% of all duplications.^1)^ Symptoms are often nonspecific, such as abdominal pain or pancreatitis, and diagnosis can be delayed.^2,3)^ Preoperative diagnosis is important for selecting an appropriate surgical strategy. EUS is particularly useful because the demonstration of a characteristic double-layered wall can help distinguish duodenal duplication from other cystic lesions.^4)^

Complete resection is generally recommended because of the risks of recurrent symptoms, pancreatitis, and, although rare, malignant transformation. However, lesions adjacent to the bile or pancreatic ducts may require pancreaticoduodenectomy, which is highly invasive in children. Minimally invasive alternatives, such as laparoscopic mucosal resection, have recently been proposed.^5)^

We report a 5-year-old girl with a duodenal duplication who was treated successfully with laparoscopic mucosal resection using intraoperative ultrasonography, emphasizing its feasibility as a safe, less invasive option.

## CASE PRESENTATION

**Fig. 1:**
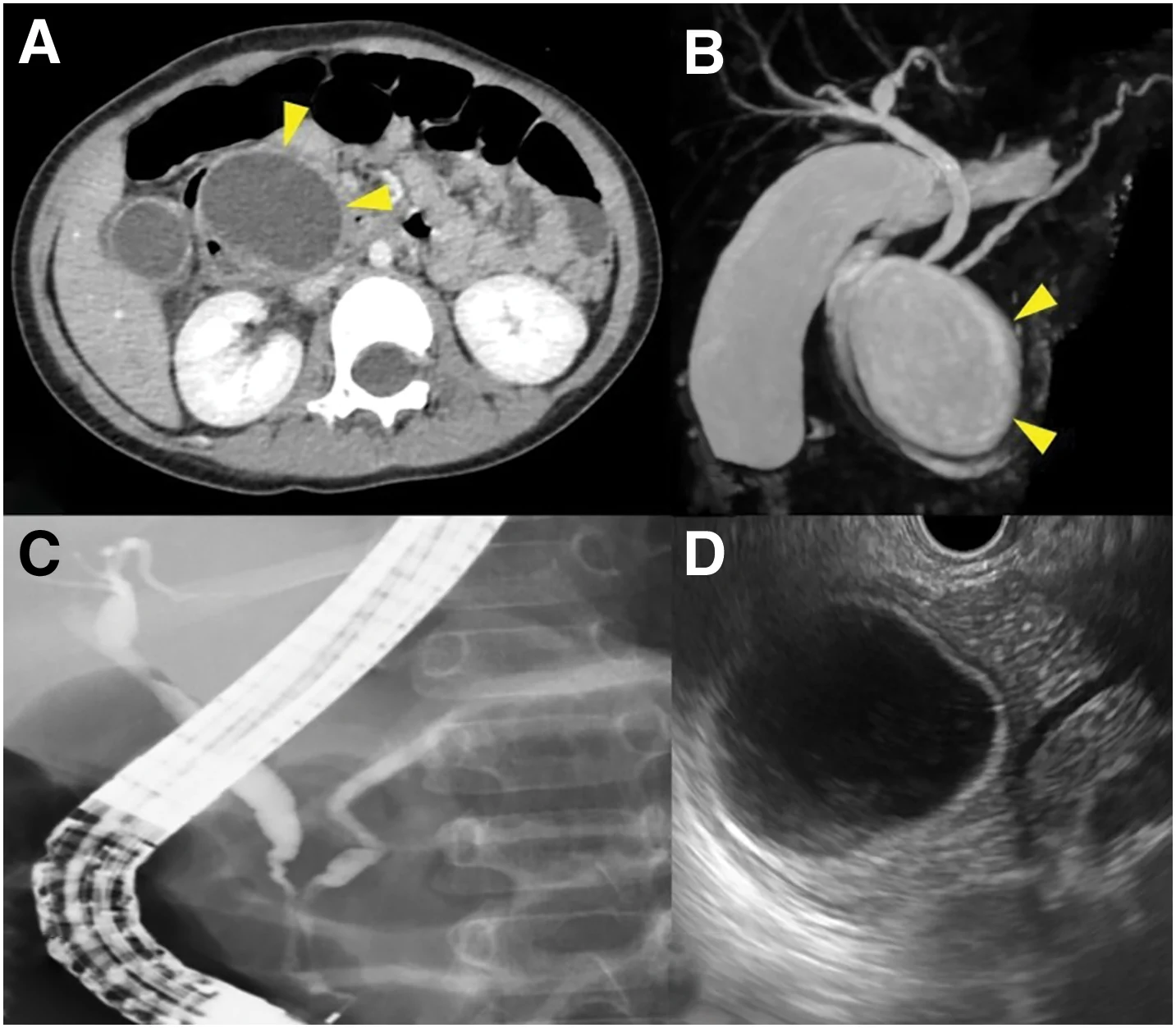
Preoperative imaging. (**A**, **B**) CT and MRCP: A cystic lesion was observed in the region of the pancreatic head (arrowheads). (**C**) ERCP: The cystic lesion did not communicate with the main pancreatic or bile ducts. No evidence of a pancreaticobiliary maljunction was observed. (**D**) EUS: The cyst wall exhibited layered structures with differing echogenicities, and mucinous contents were observed within the lesion. Continuity within the pancreas was not clearly identified, suggesting that the lesion likely originated outside the pancreas. ERCP, endoscopic retrograde cholangiopancreatography; EUS, endoscopic ultrasonography; MRCP, magnetic resonance cholangiopancreatography

A 5-year-old girl presented with abdominal pain and vomiting, with no medical comorbidities or significant family history. Contrast-enhanced CT and MRCP revealed a cystic lesion in the pancreatic head (**Fig. 1A**, **1B**), and the patient was referred to our hospital for further evaluation. The initial differential diagnoses included pancreatic tumors, pancreatic pseudocysts, type III choledochal cysts, and lymphangiomas. ERCP and EUS showed no evidence of pancreaticobiliary maljunction and no connection between the pancreatic duct and the cystic lesion (**Fig. 1C**). Based on these findings, a solid pseudopapillary neoplasm was initially suspected. Total bilirubin was normal (0.3 mg/dL), while liver enzymes were mildly elevated (aspartate aminotransferase 74 U/L; alanine aminotransferase 44 U/L). The cystic lesion caused mild compression of the distal common bile duct, resulting in slight biliary dilatation. Therefore, a biliary stent was placed, and bile analysis revealed an amylase level of 581 U/L. Her abdominal pain improved and oral intake resumed after stenting; however, the patient subsequently developed pancreatitis, with a serum amylase level of 2173 U/L. This was considered to have resulted from impaired pancreatic juice drainage due to compression of the papilla of Vater or the pancreatic duct by the lesion. Upon reevaluation of the imaging findings, the possibility of duodenal duplication was raised. Repeat EUS demonstrated that the cyst was extrapancreatic in origin, with a characteristic double-layered wall, confirming the diagnosis of a duodenal duplication (**Fig. 1D**). Because intermittent abdominal pain persisted and recurrent pancreatitis was suspected, early surgical intervention was planned.

**Fig. 2:**
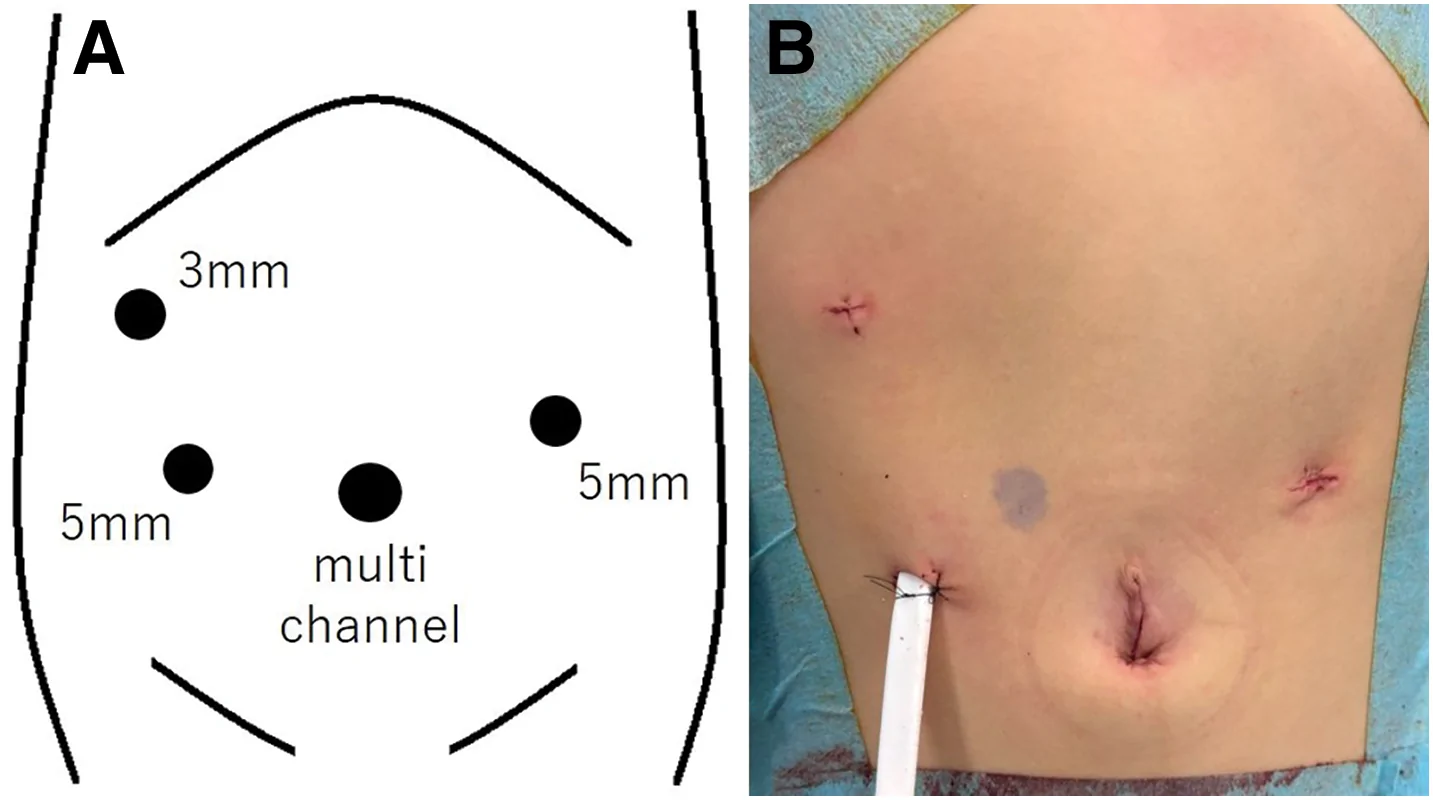
Port schema and postoperative photograph. (**A**) A multichannel port (camera) was placed at the umbilicus, two 5-mm ports (for the surgeon) were placed on the left and right sides, and a 3-mm port (for the assistant) was placed on the right upper abdomen. (**B**) A drain was placed through the right port site.

**Fig. 3:**
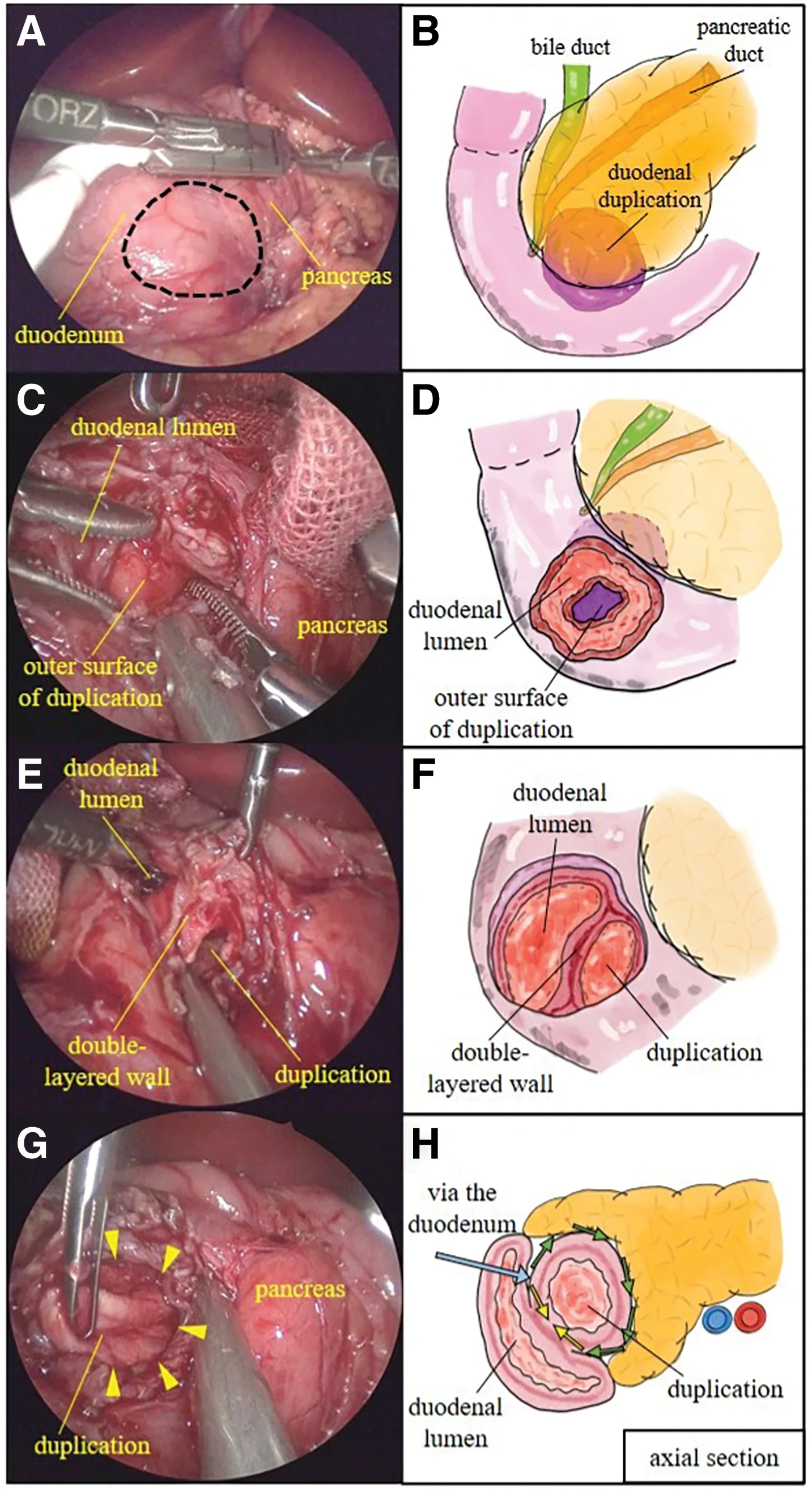
Intraoperative findings and corresponding operative schematics. (**A**, **B**) The cystic lesion was not visible on the serosal surface of either the duodenum or the pancreas (**A**). The lesion, localized by intraoperative laparoscopic ultrasonography, was indicated by the black dotted line in **A**; the corresponding anatomical relationship was illustrated schematically in **B**. (**C**, **D**) After the native duodenal lumen was opened, the outer surface of the duplication was identified under ultrasonographic guidance and mobilized from the surrounding duodenal wall (**C**), as illustrated schematically in **D**. (**E**, **F**) Opening the duplication revealed a lumen lined with duodenal mucosa (**E**). At the boundary between the duplication and the native duodenum, a double-layered wall representing a shared muscular layer was identified, confirming the intraoperative diagnosis of duodenal duplication, as illustrated schematically in **F**. (**G**, **H**) On the pancreatic side, dissection was performed along the plane between the duplication and the pancreas, allowing full-thickness excision of the duplication (yellow arrowheads in **G** and green arrows in **H**). At the shared wall with the native duodenum, only the mucosal layer of the duplication was resected while preserving the shared muscular layer (yellow arrows in **H**).

The operation was initiated with the patient in the spread-leg position. The umbilicus was incised using a Benz incision,^6)^ and a multichannel port with three 5-mm ports was inserted through the incision (**Fig. 2A**). The other ports were inserted as shown in **Fig. 2**. First, the transverse colon was mobilized to expose the duodenum, followed by Kocherization. Because the cystic lesion could not be identified on the duodenal surface, intraoperative laparoscopic ultrasonography (L51K probe compatible with the ARIETTA series, FUJIFILM, Tokyo, Japan) was used to confirm its location (**Fig. 3A**, **3B**). A cystic lesion was observed between the duodenum and the pancreatic head, consistent with the preoperative imaging. Next, a transverse incision was made on the anterior wall of the second portion of the duodenum caudal to the papilla of Vater to open the true lumen, and the papilla was identified intraluminally. Using intraoperative laparoscopic ultrasonography from within the duodenal lumen, the cystic lesion was re-localized, and the duodenal incision was extended to expose its outer surface (**Fig. 3C**, **3D**). The plane between the cystic lesion and the duodenal wall was dissected, and it was mobilized into the operative field. The cyst lumen was opened, revealing duodenal mucosa. A shared muscular layer with the native duodenum was also identified as a double-layered wall, confirming the intraoperative diagnosis of duodenal duplication (**Fig. 3E**, **3F**). Dissection along the plane between the duplication and the pancreas enabled full-thickness excision of the duplication. At the wall shared with the native duodenum, only the mucosa was removed while preserving the muscular layer (**Fig. 3G**, **3H**). The lesion was successfully removed, and the duodenum was closed transversely. The biliary stent remained in place during surgery. A drain was placed adjacent to the resection site, and the operation was completed without placement of a trans-anastomotic tube. The key steps of this procedure are shown in Additional file (**Video 1**).

**Fig. 4:**
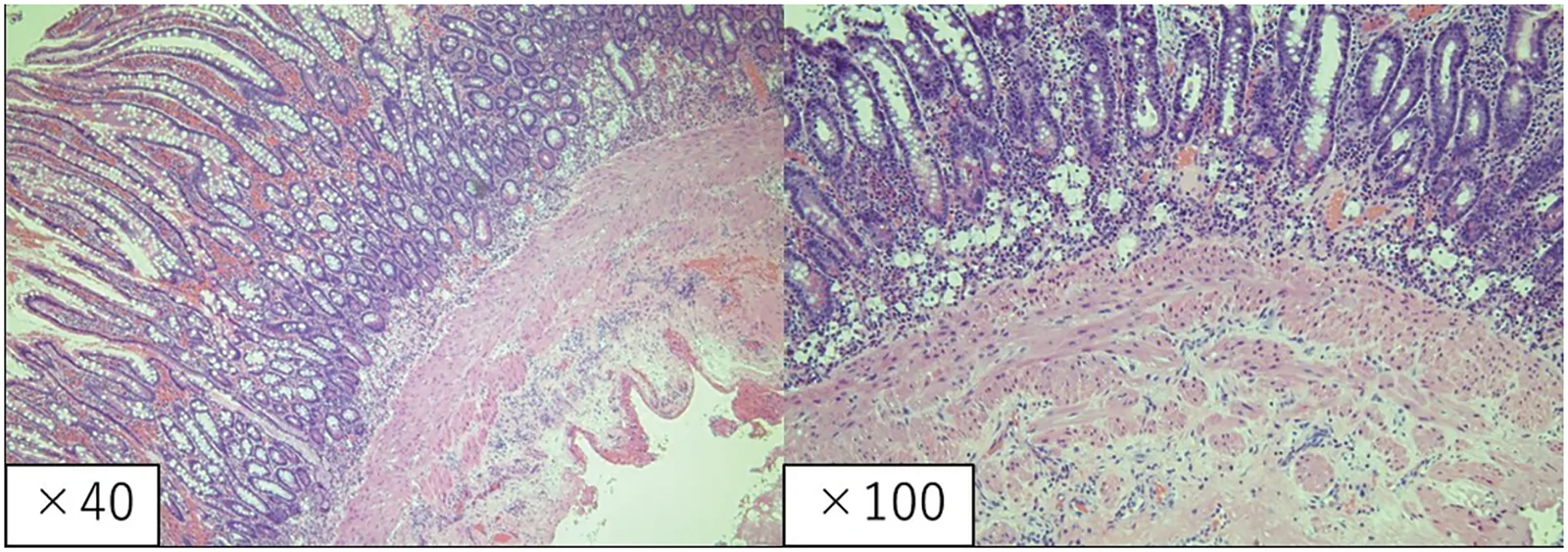
Pathological findings (hematoxylin-eosin staining). Mucosal tissue resembling the duodenum and smooth muscle tissue were observed, consistent with the findings of duodenal duplication. No evidence of malignancy was identified.

Pathological examination revealed mucosal tissue resembling duodenal mucosa and smooth muscle tissue, consistent with duodenal duplication. No evidence of malignancy was identified (**Fig. 4**).

Postoperatively, the patient developed a pancreatic fistula due to peripheral pancreatic duct injury, which required temporary pancreatic duct stenting. The patient was discharged on POD 19, and the pancreatic stent was removed 80 days after placement. The biliary stent was also removed during the same procedure. The patient has remained asymptomatic under follow-up for 1 year.

## DISCUSSION

Alimentary tract duplications are congenital anomalies that may occur anywhere along the gastrointestinal tract, with a reported incidence of approximately 1 in 4000–5000 live births. Duodenal duplications are particularly rare, accounting for approximately 6% of all alimentary tract duplications.^1)^ Based on these reported figures, the incidence of duodenal duplication can be estimated to be approximately 1.3 cases per 100000 live births. However, because some cases remain asymptomatic until adulthood, the actual frequency of diagnosis during childhood may be even lower.

Duodenal duplication often presents with nonspecific symptoms, such as abdominal pain, vomiting, jaundice, or pancreatitis.^2,3)^ In the meta-analysis by Chen et al., abdominal pain was the most common symptom and pancreatitis was the most frequent complication, indicating that this entity should be considered in the differential diagnosis of cystic lesions around the pancreatic head and periampullary region.^2)^ Preoperative diagnosis is often difficult because these lesions may mimic pancreatic pseudocysts, choledochal cysts, lymphangiomas, or pancreatic tumors. In this setting, EUS is particularly valuable because demonstration of a characteristic double-layered wall can help establish the diagnosis and clarify the lesion’s origin when CT or MRCP is inconclusive.^3,4)^ Although no communication was demonstrated by preoperative ERCP or EUS, the bile-like fluid observed after opening the duplication may have entered through a minute communication with the native duodenal lumen or pancreaticobiliary system that was below the resolution of preoperative imaging and was not macroscopically identifiable during surgery.

With regard to treatment, complete resection is generally recommended because of the risk of persistent symptoms and malignant transformation.^3,7)^ However, when a lesion is located adjacent to the bile or pancreatic duct, particularly the pancreatic head, complete removal requires pancreaticoduodenectomy. Given the high morbidity associated with this procedure in pediatric patients, less invasive alternatives are desirable.^3)^

Duodenal duplication can be managed by several different approaches depending on the lesion’s location and its relationship to the native duodenum, papilla of Vater, and pancreaticobiliary ducts. Endoscopic marsupialization or fenestration has been reported as a less invasive option for selected intraluminal lesions, but this approach may leave residual mucosa and is less suitable for extraluminal lesions.^5,8)^ In contrast, surgical treatment remains the mainstay for extraluminal lesions, particularly those near the pancreatic head. Recent reports have described complete laparoscopic resection and robot-assisted transduodenal excision, suggesting that minimally invasive surgery can provide organ-preserving alternatives to radical resection in selected patients.^5,9,10)^ Thus, the operative approach should be individualized according to lesion location and the feasibility of complete mucosal removal without undue morbidity.

In our case, the patient was 5 years old, and long-term concerns such as symptom recurrence or malignant transformation were taken into account. Therefore, we aimed for complete resection whenever feasible. Intraoperatively, a dissection plane between the lesion and the pancreas was preserved, which enabled full-thickness excision on the pancreatic side. We acknowledge that dissection near the pancreatic head may increase the risk of pancreatic fistula; nevertheless, we chose full-thickness excision because a clear and safe dissection plane was identified intraoperatively, allowing controlled separation with minimal traction. We also considered mucosal stripping on the pancreatic side; however, given the patient’s young age and the potential long-term risk of recurrence and malignant transformation,^7)^ we prioritized complete excision when safely achievable. Additionally, the lesion was located dorsally on the pancreatic side of the duodenum and was not visible from the serosal surface. Therefore, after opening the true duodenal lumen, intraoperative laparoscopic ultrasonography was used to precisely localize the lesion and facilitate safe transduodenal access. This approach allowed tailored resection while preserving the surrounding structures and avoiding more invasive surgery. LECS could also be considered as an organ-preserving option. In the present case, however, its additional benefit was considered limited. The lesion was predominantly extraluminal and located on the pancreatic side of the duodenum; therefore, the principal technical challenges were precise localization of the lesion and identification of a safe dissection plane between the duplication and the pancreas. These challenges would not have been resolved by endoscopic visualization alone. Although endoscopy could have facilitated intraluminal orientation and identification of the papilla of Vater, intraoperative ultrasonography and direct transduodenal exposure already provided the information required to localize the lesion, identify the shared wall, and determine the appropriate extent of resection. Thus, LECS was unlikely to substantially alter the operative strategy or meaningfully reduce the risk of pancreaticobiliary injury in this case.

A key limitation of this approach is the risk of pancreatic duct injury during dissection on the pancreatic side. In the present case, although a clear dissection plane was identified intraoperatively, the patient developed a postoperative pancreatic fistula caused by injury to a peripheral pancreatic duct. This complication was successfully managed by temporary pancreatic duct stenting. This experience indicates that, even when organ-preserving laparoscopic resection is feasible, surgeons should carefully balance the extent of full-thickness excision against the risk of pancreatic duct injury. In cases in which the dissection plane is unclear or the lesion is closely adherent to the pancreatic parenchyma, mucosal stripping rather than full-thickness excision may be a safer alternative.

## CONCLUSIONS

This case suggests that laparoscopic transduodenal mucosal resection with selective full-thickness excision under intraoperative ultrasonographic guidance can provide an organ-preserving option for selected pediatric patients with duodenal duplications near the pancreatic head. However, the risk of pancreatic duct injury should be recognized, particularly when full-thickness dissection is performed on the pancreatic side.

## SUPPLEMENTARY MATERIALS

Video 1 Laparoscopic transduodenal mucosal resection with selective full-thickness excision under intraoperative ultrasonographic guidance.

## DECLARATIONS

### Availability of data and materials

The datasets supporting the conclusions of this article are included within the article.
